# Dystrophic Epidermolysis Bullosa: Secondary Disease Mechanisms and Disease Modifiers

**DOI:** 10.3389/fgene.2021.737272

**Published:** 2021-09-28

**Authors:** Alexander Nyström, Leena Bruckner-Tuderman, Dimitra Kiritsi

**Affiliations:** ^1^Department of Dermatology, Medical Faculty, Medical Center - University of Freiburg, Freiburg, Germany; ^2^Freiburg Institute for Advanced Studies, Freiburg, Germany

**Keywords:** collagen VII, fibrosis, inflammation, transforming growth factor β, skin

## Abstract

The phenotypic presentation of monogenetic diseases is determined not only by the nature of the causative mutations but also is influenced by manifold cellular, microenvironmental, and external factors. Here, heritable extracellular matrix diseases, including dystrophic epidermolysis bullosa (DEB), are no exceptions. Dystrophic epidermolysis bullosa is caused by mutations in the *COL7A1* gene encoding collagen VII. Deficiency of collagen VII leads to skin and mucosal fragility, which progresses from skin blistering to severe fibrosis and cancer. Clinical and pre-clinical studies suggest that targeting of secondary disease mechanisms or employment of natural disease modifiers can alleviate DEB severity and progression. However, since many of these mechanisms are needed for tissue homeostasis, informed, selective targeting is essential for safe and efficacious treatment. Here, we discuss a selection of key disease modifiers and modifying processes active in DEB, summarize the still scattered knowledge of them, and reflect on ways forward toward their utilization for symptom-relief or enhancement of curative therapies.

## Introduction

Epidermolysis bullosa (EB) comprises a group of genetic disorders manifested by mechanically induced blistering and fragility of the skin and other stratified epithelia. Over 20 genes have been described to be causative of EB and related skin fragility disorders (Has et al., 2020). These span genes encoding intracellular transporters, *via* cytoskeletal proteins and integrin receptors, to extracellular matrix (ECM) proteins. Depending on the level of separation in the skin, EB is divided into four main types: Epidermolysis bullosa simplex with blistering occurring in the epidermal basal keratinocyte layer, junctional EB with blistering within the lamina lucida of the epidermal basement membrane, dystrophic EB (DEB) with blistering below the epidermal basement membrane, and Kindler EB with blistering occurring in all layers (Bardhan et al., 2020).

This mini-review will focus on DEB, which can be inherited in a dominant or a recessive (RDEB) manner (Bardhan et al., 2020). Dystrophic epidermolysis bullosa is primarily caused by genetic loss of function or abundance of collagen VII, encoded by the *COL7A1* gene (Has et al., 2018), but a number of secondary, molecular and cellular, events modify the disease phenotype.

## Collagen Vii

Collagen VII is a large, in parts atypical collagen, which attaches the epidermal basement membrane to the dermal ECM. It is synthesized as pro-collagen VII consisting of three pro-α1 chains that fold into one pro-collagen VII molecule. The molecule has sizable N-terminal non-collagenous 1 (NC1) domains, followed by an extended collagenous domain, and ends with minor NC2 domains at the C-terminus (Has et al., 2018). Although minor, the NC2 domain is essential for intermolecular assembly (Bruckner-Tuderman et al., 1995; Chen et al., 2001). Extracellularly in the skin, pro-collagen VII molecules align as antiparallel dimers, which laterally aggregate to form anchoring fibrils (Chung and Uitto, 2010). The antiparallel dimer formation occurs over the NC2 domain, and in a stretched configuration, anchoring fibrils consequently contain NC1 domains at both ends and the NC2 domains in the middle. Proper removal of a large part of the NC2 domain is essential for stable anchoring fibril formation (Bruckner-Tuderman et al., 1995; Chen et al., 2001). Astacin-like proteases BMP-1/mTLD and meprins have been shown to cut pro-collagen VII to collagen VII (Rattenholl et al., 2002; Moali et al., 2005; Kruppa et al., 2021).

In the skin, epidermal keratinocytes and papillary dermal fibroblasts produce collagen VII and contribute to its deposition at the dermal-epidermal junction (DEJ; Twaroski et al., 2019). The NC1 domains are generally positioned in the epidermal basement membrane and the collagenous domains loop down into the superficial papillary ECM (Has et al., 2018). Anchoring fibrils provide skin stability by attaching the epidermal basement membrane, *via* high-affinity interactions of their collagen VII NC1 domains with laminin-332 and collagen IV, and simultaneous binding to collagen fibrils in the papillary dermis (Chen et al., 1997, 1999; Brittingham et al., 2006; Villone et al., 2008). It appears that the sixth or seventh fibronectin type III-like domain in the NC1 domain harbors the major binding sites for laminin-332, collagen IV, and even the weaker interaction partner thrombospondin-1 (Aho and Uitto, 1998; Chen et al., 1999; Brittingham et al., 2006; Atanasova et al., 2019).

For the correct function, deposition and stability of collagen VII and anchoring fibrils posttranslational modifications are needed. Currently, there is an insufficient understanding on the exact role of these modifications. Collagen VII appears to be a substrate of the cross-linking enzyme transglutaminase 2 (TGM2), and TGM2-mediated crosslink formation may stabilize anchoring fibrils (Raghunath et al., 1996; Küttner et al., 2014). In addition, collagen VII has been suggested to be modified by the multi-functional enzyme lysyl hydroxylase 3 (LH3) also known as pro-collagen-lysine, 2-oxoglutarate 5-dioxygenase 3 (Watt et al., 2015; Vahidnezhad et al., 2019). Lysyl hydroxylase 3 both hydroxylates lysyl residues and then further O-glycosylates these (Salo et al., 2006; Risteli et al., 2009). Lysyl hydroxylase 3 deficiency is linked to altered deposition and reduced functionality of multiple tissue-stabilizing collagens, including collagen VII. Interestingly, skin blistering in LH3-deficient skin shows similarities to that of collagen VII-deficient RDEB skin (Salo et al., 2008; Vahidnezhad et al., 2019). In addition to posttranslational modifications, coordinated production and deposition of collagen VII by both keratinocytes and fibroblasts have been indicated to facilitate anchoring fibril assembly (Supp et al., 2019). Collectively, the complex synthesis and modification of collagen VII, the contribution of multiple cellular sources to anchoring fibril formation and the incomplete understanding of these processes, pose challenges for RNA, gene, protein, or cellular therapies aiming to restore collagen VII and anchoring fibrils in RDEB.

## Genotype-Phenotype Correlations

**Figure 1:**
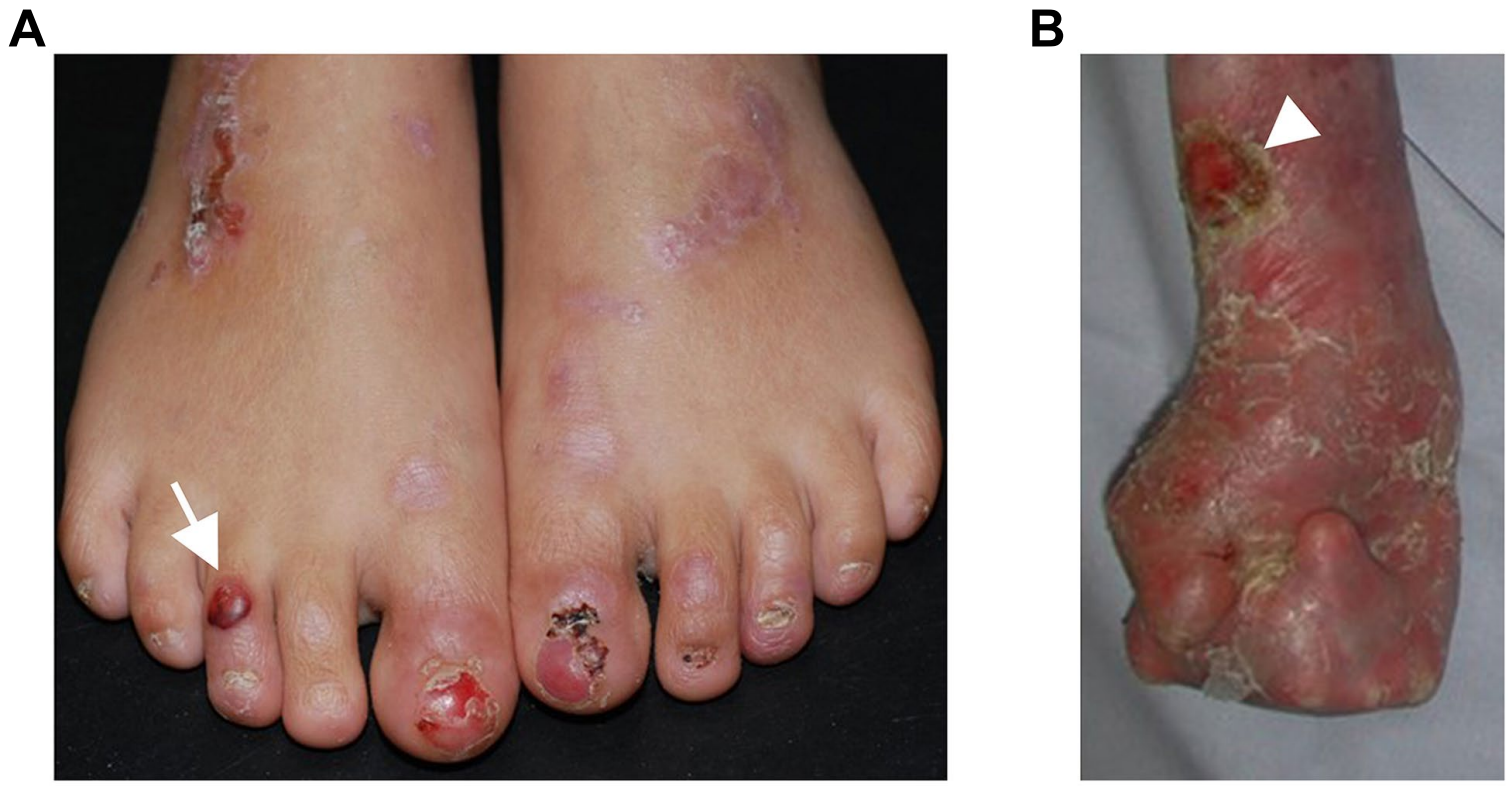
Phenotypic variability in dystrophic epidermolysis bullosa. **(A)** A 6-year-old patient with recessive DEB and residual collagen VII expression has blisters (arrow) and erosions on the feet. The nails are dystrophic or absent, scars and erythematous maculae occur after healing of the lesions. **(B)** A 27-year-old patient with severe RDEB and complete lack of collagen VII expression suffers from severe skin fragility, chronic wounds (arrowhead), and pronounced, progressive fibrosis of the skin, resulting in mitten deformities. The skin appears inflamed with redness and crusts in areas of healed blisters.

More than 1,000 distinct mutations have been reported to cause DEB. The most severe phenotypes are associated with *COL7A1* mutations causing complete loss of translated collagen VII. However, the genotype–phenotype correlations are not completely clear and there is a vast phenotypic variability (Figure 1). There are cases with sole nail dystrophy or mild localized disease. These are mostly associated with glycine substitutions in the collagenous domain (Dang and Murrell, 2008). Patients might suffer from an inversa phenotype, with skin fragility mostly in the flexural skin areas and the mucosa. This seems to be caused by recessive arginine and glycine substitutions in the collagenous domain (van den Akker et al., 2011), and the hypothesis was proposed that the higher temperature in the body flexures impairs stability of the glycine-substituted collagen VII, leading to skin lesions in these specific areas (van den Akker et al., 2011). The rare subtype, DEB pruriginosa, clinically characterized by intensively itchy, hypertrophic, prurigo-like papules, and nodules, is in more than 50% of the cases associated with glycine substitutions, followed by in-frame skipping mutations in around 30% (Kim et al., 2015). These data highlight that the type of mutation but also its position within collagen VII are phenotypic determinants (Dang and Murrell, 2008) with a broad range of cutaneous manifestations. Further, the nature of amino acid change for a given position is important, as shown by the different phenotypes caused by mutations in the same position (Almaani et al., 2011). Nonetheless, even in patients with the same *COL7A1* mutations the phenotypes might differ, as shown by studies in siblings (Hovnanian et al., 1997; Bodemer et al., 2003; Titeux et al., 2008; Odorisio et al., 2014), disclosing that in addition to the causative mutations, other genetic, epigenetic, microenvironmental, and environmental factors contribute to the phenotype. As discussed below, these factors remain limitedly known. A better understanding of them is important for improved prognostication of the disease severity and therapeutic exploitation for disease-modifying therapies. Pathological *COL7A1* mutations could also be used to provide insights on the collagen VII interactome; however, it should in this context be mentioned that missense mutations in known protein–protein interacting domains, such as the NC1 domain, are exceedingly rare [COL7A1(gene) - ClinVar – NCBI, 2020].

## Cutaneous Manifestations in Rdeb

The disease hallmark of RDEB is skin fragility, which manifests with the mechanically induced development of blisters and erosions, especially in trauma-prone skin areas, such as hands, feet, and knees (Figure 1). Acute or chronic wounds occur at different body sites with itch and pain as a consequence (Solis et al., 2021). Also the mucosa and adnexal structures of the skin might be affected, with features of intraoral soft-tissue involvement and dental abnormalities (Krämer et al., 2020), nail dystrophy, and secondary alopecia. Ocular involvement encompasses corneal erosions and subsequent scarring, as well as eye lid erosions, followed by ectropion or symblephara (Figueira et al., 2010). These may result in severe impairment of visual acuity.

The cutaneous and mucosal blisters heal with scarring. At sites, meeting frequent mechanical and frictional challenges, typically the extremities, fibrosis driven by a chronic state of injury and inflammation leads to joint contractures and webbing of fingers and toes and the formation pseudosyndactylies – so called mitten deformities – allowing only limited function and mobility (Figure 1B).

The intermittent blistering, impaired wound healing with inflammation, high bacterial load, wound infections, and subsequent fibrosis of the skin are considered to be main events creating a microenvironment that promotes mutagenesis of keratinocytes through, e.g., activation of cell-intrinsic DNA editing enzymes, such as the APOBECs (Hoste et al., 2015; Cho et al., 2018). These events also establish a stiffened and hyper-vascularized environment, facilitating the growth, progression, and metastasis of established squamous cell carcinomas (SCCs; Martins et al., 2016; Mittapalli et al., 2016; Föll et al., 2017; Condorelli et al., 2019). These arise already in early adulthood and are the main cause of death in patients with severe generalized RDEB (Montaudié et al., 2016). Notably, in that specific patient, population more than 90% will have developed an SCC by the age of 55years and have a 78% cumulative risk of death from metastatic SCCs (Fine et al., 2009).

## Extracutaneous Manifestations in Rdeb

Besides the disease manifestations in skin and mucosa, complications arise in other organs. Such extracutaneous manifestations are more prominent in patients with the severe subtypes of RDEB. The perpetual cycles of trauma-induced blistering of the esophagus result in stenoses, requiring dilatations, and/or gastrostoma to ensure proper food intake. Consequences are anemia, partially due to iron deficiency, deficiencies in vitamins and minerals, and severe failure to thrive (Reimer et al., 2020). With progressive disease, connected to the systemic impact of cutaneous wounding and inflammation, internal organs can become affected. Renal parenchymal disease might arise, linked to amyloidosis or the autoimmune disease IgA nephritis (Bardhan et al., 2020). A subset of individuals with RDEB develops life-threatening dilated cardiomyopathy at an early age (Fine et al., 2008). A less considered change is external auditory canal stenosis, which may result in hearing loss (Brown et al., 2017).

### Immune Anomalies in RDEB

People with RDEB have elevated bacterial colonization of wounds compared to non-RDEB individuals with large, chronic wounds (Levin et al., 2021), with *Staphylococcus aureus* and *Streptococcus pyogenes* being the most common colonizers (Fuentes et al., 2018; Levin et al., 2021). In addition, RDEB wounds and skin show a reduced diversity of their bacterial microbiome (Bar et al., 2021). Our studies of RDEB patients and RDEB mouse models indicate that the increased susceptibility to bacterial infections is not only associated with wounding, but also to a large extent due to loss of collagen VII from secondary lymphoid organs (Nyström et al., 2018). One mechanism making people with RDEB unable to respond appropriately to bacterial challenges is through loss of interactions between collagen VII and the ECM protein cochlin in the conduits of secondary lymphoid organs. Upon bacterial challenges, systemic danger signals, through, e.g., TNF, increase expression of aggrecanase 1 and 2 in the spleen and other secondary lymphoid organs. Aggrecanases in turn release the N-terminal LCCL domain of cochlin into the circulation. The LCCL domain promotes activation and boosting of antibacterial immunity exerted by innate immune cells (Py et al., 2013). Collagen VII binds cochlin with high affinity and appears to serve as its major anchor in secondary lymphoid organs. Loss of collagen VII evokes a dramatic loss of cochlin from these organs and, thus, an inability to mount a cochlin LCCL domain-mediated innate immune response toward bacteria (Nyström et al., 2018).

Intriguingly, besides the abnormalities in the innate immune system, also anomalies in adaptive immunity including presence of autoantibodies against several proteins of the DEJ have been reported (see below; Tampoia et al., 2013; Annicchiarico et al., 2015; Esposito et al., 2016). If these changes are bystanders of chronic injury or if they represent active participants in disease pathogenesis needs to be elucidated; however, observation of alleviation of disease manifestations in RDEB patients receiving various forms of immunosuppressive or immunomodulatory treatment – mild to harsh (Wagner et al., 2010; Petrof et al., 2015; Ebens et al., 2019) – could suggest active participation to disease.

## Disease Modifiers

There is a general tendency that higher levels of collagen VII expression positively correlate with milder disease in RDEB (van den Akker et al., 2009). Upon closer inspection, this tendency is not constant and the mechanisms regulating disease severity are not well understood. Unexpectedly, the same mutation combinations even in the same family can result in widely disparate disease severity (Hovnanian et al., 1997), indicating the presence of strong environmental, genetic, and epigenetic modifiers of disease in RDEB. The detailed nature of such modifiers remains for most elusive or not definitely proven, reflecting the challenges in generating robust data and statistically assessing these in a small subset of a rare disease (Kern et al., 2009). Below, we discuss molecular and cellular players that are thought to act as disease modifiers in DEB.

### Proteolytic Activities

**Figure 2:**
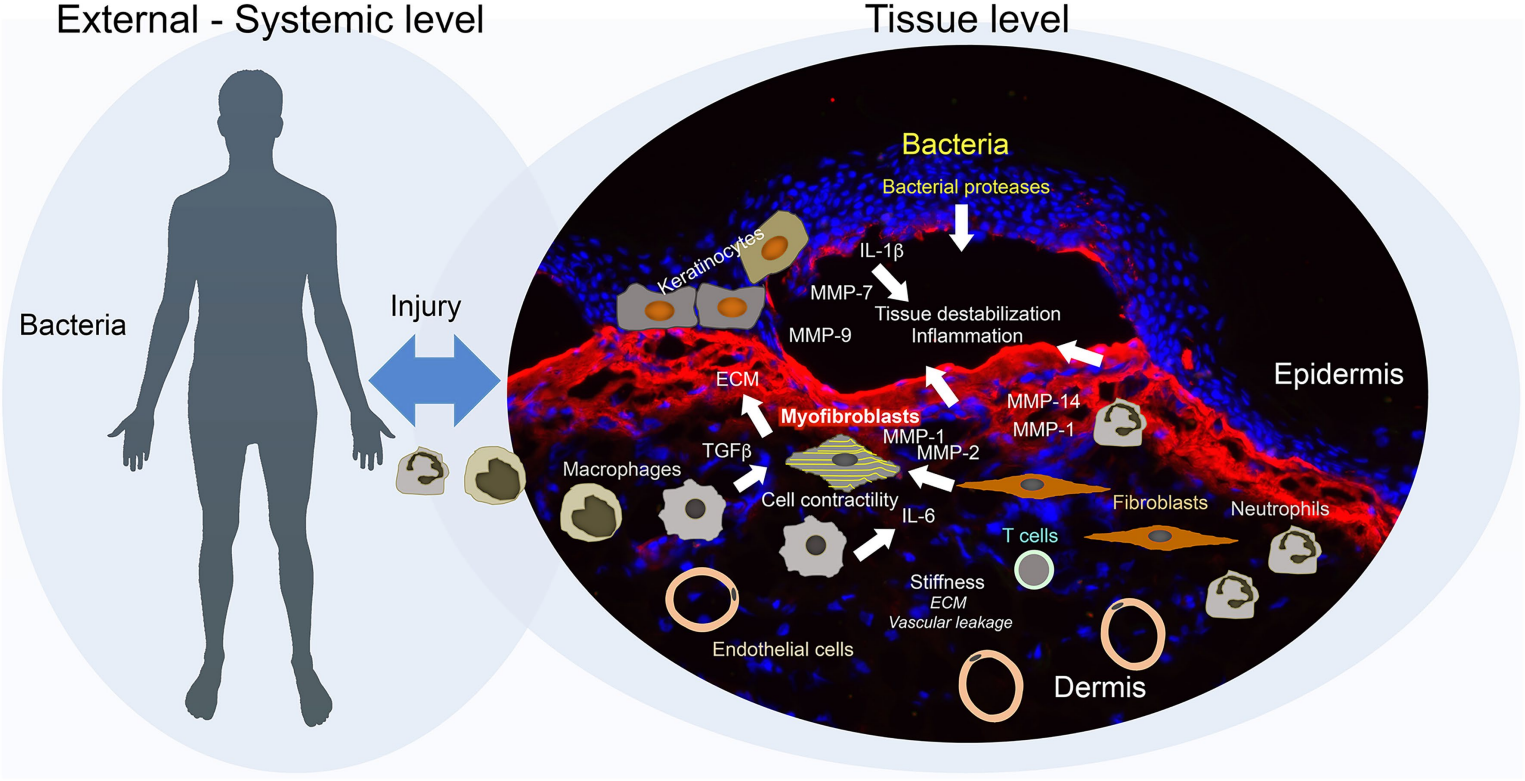
Secondary disease mechanisms and potential disease modifiers in dystrophic epidermolysis bullosa (DEB). Disease progression in DEB is jointly driven by external and tissue-intrinsic factors. Frictional and mechanical challenges induce blistering of a pre-destabilized tissue, this together with high bacterial load trigger an exaggerated inflammatory and wound healing response. Bacteria may promote direct destruction of tissue through release of bacterial proteases. Epidermal keratinocytes, activated by impaired adhesion, injury, and inflammation, increase production of proteases, including matrix metalloproteinases (MMPs), which further evoke tissue degradation and inflammation. In addition, the keratinocytes secrete pro-inflammatory factors and trigger activation of dermal fibroblasts. Inflammatory cells – inflammatory macrophages and neutrophils – promote tissue degradation through secretion of MMPs and elastase, and activation of fibroblasts through release of, e.g., TGFß and IL-6. Activated fibroblasts respond by increasing production of provisional and interstitial extracellular matrix (ECM) and altering the ECM architecture to a stiffer structure. Vascular leakage enhanced by the hyper-vascularized tissue may cause a transient increase in stiffness triggering stiffness response in fibroblasts. Increasing stiffness and TGFβ, which is released by multiple cell types – keratinocytes, fibroblasts, inflammatory cells, and platelets – and activated by several mechanisms – integrins, proteases, thrombospondin-1, and ROS – promote conversion of fibroblasts to contractile myofibroblasts. With time, adaptive immune activation occurs which may further enhance chronic inflammation and injury response. Collectively, these multifaceted mechanisms promote the establishment of a fragile, fibrotic, and contractile tissue that supports the growth of squamous cell carcinomas.

Dystrophic epidermolysis bullosa has been associated with increased proteolytic activity in epidermal and dermal microenvironments (Eisen, 1969; Lazarus, 1972). Human and murine RDEB skin and cultured RDEB skin-derived keratinocytes and fibroblasts display alterations in the expression and the activity of several proteases and inhibitors, including matrix metalloproteinase (MMP)-1, MMP-2, MMP-3, MMP-9, MMP-7, MMP-13, MMP-14, TIMP-1, TIMP-3, cathepsin B and Z, meprins, and fetuin B (Valle and Bauer, 1980; Winberg et al., 1989; Bodemer et al., 2003; Küttner et al., 2013; Liao et al., 2018; Thriene et al., 2018; Akasaka et al., 2021; Figure 2).

Dysregulation of lysosomal proteases occurs both in RDEB keratinocytes and fibroblasts (Küttner et al., 2013; Thriene et al., 2018). These impair cellular fitness by affecting autophagy and causing a senescent, pro-inflammatory phenotype with limited regenerative abilities (Küttner et al., 2013; Thriene et al., 2018; Berberich et al., 2020).

Matrix metalloproteinases, especially the collagenase and gelatinases, have been the focus of most investigations on elevated protease activity in DEB. Early studies, before the genetic cause of RDEB had been disclosed, revealed increased collagenase and gelatinase activity in DEB skin blisters (Eisen, 1969; Lazarus, 1972), but also increased activities in non-blistered DEB skin were suggested (Eisen, 1969). It was concluded that “the increased amounts of collagenase might perpetuate the blistering and scarring by degrading the connective tissue” (Lazarus, 1972). Based on findings that phenytoin reduced collagenase expression, it was repurposed to treat RDEB (Bauer et al., 1980). First results were promising, indicating reduction of blistering (Bauer et al., 1980). However, a subsequent, larger study could not confirm a benefit (Caldwell-Brown et al., 1992) and the use of phenytoin for RDEB was consequently largely abandoned.

Interestingly, dysregulated collagenase and gelatinase activity are maintained in cultured RDEB keratinocytes and fibroblasts (Bauer and Eisen, 1978; Valle and Bauer, 1980; Akasaka et al., 2021), which suggests long-lasting intrinsic changes attributed to genetics, epigenetics, cellular responsiveness, or cellular memory. However, this is not a general phenomenon observed in all studies and whether or not these activities are heighted compared to donor cells appear to depend on donors and culture conditions (Winberg et al., 1989; Küttner et al., 2013; Akasaka et al., 2021).

The variability in collagenase and gelatinase activity between individuals with DEB, together with the fact that they may degrade collagen VII, resulted in the hypothesis that MMP activity could be a disease modifier for DEB (Bodemer et al., 2003). In three brothers with RDEB with dramatically discordant severity, the abundance of MMP-1, −2, −3, and−9 was increased in both blisters and non-blistered skin, as compared to healthy control skin. Skin from the intermediately affected brother showed the highest MMP-2 and -9 activity. However, the abundance of MMP-1 was greatest in the most severely affected brother and this correlated with MMP-1 activity being uniquely detected in his blistered skin. Building on these studies, a subsequent investigation identified a SNP in the *MMP-1* promoter associated with higher *MMP-1* expression in the more severely affected brothers and further linked this SNP to more severe RDEB in a larger cohort (Titeux et al., 2008). Higher frequency of the specific *MMP-1* SNP was also detected in RDEB compared to healthy controls in another study (Almaani et al., 2009). Analysis of a larger DEB cohort confirmed higher frequency of the *MMP-1* SNP in RDEB, however, could not make a correlation between the *MMP-1* SNP and more severe RDEB (Kern et al., 2009).

Early clinical trials on cancer treatment with MMP inhibitors were unsuccessful, which was due to multiple factors including low selectivity of the inhibitors, challenges with bioavailability and insufficient understanding of MMP function, and their interconnectivity in the protease web (Winer et al., 2018; Fields, 2019). Progress of optimized inhibitor synthesis and overall enhanced knowledge of protease biology has created a renewed interest in MMP targeting in multiple diseases from autoimmune diseases to cancers (Winer et al., 2018; Fields, 2019). Research for the application of MMP targeting for DEB could ride this wave. However, to avoid repeating past failures related to uninformed targeting, it is important to first gain a comprehensive understanding of the protease web in DEB and its dysregulation, as proteases are active on both sides of the disease spectrum – they are degenerative and may promote inflammation but also essential for tissue regeneration and inflammatory homeostasis. Apart from their processing and degrading the ECM, they are essential regulators of transforming growth factor (TGF)β activity, inflammation, and angiogenesis – three other potential phenotype modifiers in RDEB.

### TGFβ Activity

Increased TGFβ activity has since long been associated with fibrotic diseases (Kim et al., 2018). In a chronically injured tissue setting as in RDEB, it is contributed by both activated tissue-resident cells as well as inflammatory cells and platelets (Kiritsi and Nyström, 2018; Figure 2). TGFβ is a pleiotropic cytokine, part of the larger TGFβ superfamily. Humans have three isoforms TGFβ1-3, which signal through TGFβ receptors type I and II, and in the canonical pathway phosphorylate SMAD-2/3, which in a complex together with SMAD4 translocate to nucleus and promote gene expression. Non-canonical TGFβ signaling also occurs, activating multiple other pathways including JNK, MAPK, and AKT. TGFβ has a variety of essential roles in organ and tissue homeostasis. It exerts immunoregulatory actions, stimulates epithelial cell mobility, epithelial-to-mesenchymal transition, activation of fibroblasts, and production of ECM proteins (Ramirez et al., 2014; Hinz, 2015; Sanjabi et al., 2017; Figure 2).

TGFβ is secreted in a latent form that needs to be activated to allow for signaling through its receptors (Kim et al., 2018). Multiple mechanisms can activate TGFβ; the reliance on these mechanisms differs depending on the context and the TGFβ isoforms. These mechanisms include activation through application of mechanical forces to the latency-associated pro-peptide *via* RGD-binding integrins, breaking of chemical bonds *via* reactive oxygen species, induction of sterical shifts *via* binding to the ECM protein thrombospondin-1, proteolytic release *via* proteases including MMPs, and on lymphocytes *via* binding to GARP (Wang et al., 2012, 2017; Kim et al., 2018).

Recent data suggest an intricate relationship between collagen VII and TGFβ. Collagen VII can downregulate TGFβ activity *in vitro* and *in vivo* reducing scarring and it also seems to limit conversion of latent to active TGFβ (Wang et al., 2013; Nyström et al., 2013b; Akasaka et al., 2021). In a viable mouse model of severe RDEB – the collagen VII hypomorphic mouse – increased abundance of TGFβ ligands was seen in fibrotic forepaws (Fritsch et al., 2008). Healing skin wounds in the same model showed elevated canonical TGFβ signaling (Nyström et al., 2013b). Interestingly, increased TGFβ ligand expression was not disclosed in heavily scarred deformities of RDEB patients (Breitenbach et al., 2015a), whereas we observed elevated levels of circulating TGFβ in RDEB, as well as tissue-bound TGFβ and increased canonical TGFβ signaling in human RDEB wounds (Nyström et al., 2015). Collectively, these results may reflect differences in the dependence of TGFβ during the onset and after establishment of fibrosis.

Cultured RDEB keratinocytes and fibroblasts display signs of increased TGFβ ligand expression and activity (Knaup et al., 2011; Küttner et al., 2013; Odorisio et al., 2014; Nyström et al., 2015; Atanasova et al., 2019). Keratinocytes contribute to fibrosis by facilitating TGFβ activation (Thriene et al., 2018; Akasaka et al., 2021). Furthermore, TGFβ activity appears to be strongly dependent on culture conditions and time (Akasaka et al., 2021). In RDEB fibroblasts and keratinocytes, RGD-binding integrins, ROS, thrombospondin-1, and MMP-2 and 9 have been suggested to participate in latent TGFβ activation (Atanasova et al., 2019; Chacón-Solano et al., 2019; Akasaka et al., 2021) and the reliance of these changes with culture time (Akasaka et al., 2021). Consequently, these processes and interactions can themselves be disease modulating.

A groundbreaking paper from Odorisio and colleagues linked TGFβ activity to disparate disease presentation in identical twins with RDEB (Odorisio et al., 2014). The twins synthesized and secreted similarly reduced levels of collagen VII, but the TGFβ activity in the skin differed. Low TGFβ activity was associated with milder disease and high TGFβ activity with a severe phenotype. Mechanistically, the TGFβ activity correlated inversely with the production of the small leucine-rich proteoglycan decorin (Odorisio et al., 2014). Decorin is a multi-functional protein with a large interactome and many biological functions assigned to its name (Neill et al., 2012). The focus of decorin in the context of the discordant RDEB phenotype was on its ability to sequester TGFβ and hide it from presentation to its cognate receptors (Yamaguchi et al., 1990; Odorisio et al., 2014). Subsequently, experimental pre-clinical support for the abilities of decorin to modify RDEB severity was obtained from systemic overexpression of decorin in collagen VII hypomorphic mice (Cianfarani et al., 2019). Expression of human wild-type decorin in newborn collagen VII hypomorphic mice significantly improved survival and reduced formation of mitten deformities of forepaws (Cianfarani et al., 2019). In addition, systemic injections of recombinant decorin fused to a wound target-seeking peptide (Järvinen and Ruoslahti, 2010) extended the life span of completely collagen VII-deficient mouse pups, reduced TGFβ ligand gene expression and markers associated with fibrosis (Pemmari et al., 2020).

TGFβ activity in RDEB is a double-edged sword – on the one hand, it increases fibrosis by activating fibroblasts and promoting ECM production, and on the other hand, it is a strong inductor of collagen VII expression. In the setting of tumors, it inhibits keratinocyte proliferation but also creates a stiffened and hypervascular microenvironment supporting tumor progression, the stiffness combined with immunosuppression may reduce anti-tumor immune surveillance (Ryynänen et al., 1991; Salmon et al., 2012; Martins et al., 2016; Mittapalli et al., 2016; Sanjabi et al., 2017). This makes direct TGFβ targeting for treatment of RDEB challenging.

In an attempt to limit TGFβ activity with a therapeutically relevant pharmacological approach, we treated collagen VII hypomorphic mice with the angiotensin II type 1 receptor antagonist losartan (Nyström et al., 2015). Losartan had in previous studies been shown to reduce TGFβ activity in fibrotic conditions, including other genetic ECM diseases (Habashi et al., 2006). RDEB fibroblasts responded to losartan by reducing pro-fibrotic activation status. Prolonged losartan treatment in adult collagen VII hypomorphic mice protected against progression of dermal fibrosis (Nyström et al., 2015). The outcome of this pre-clinical study prompted the initiation of a clinical trial testing the safety and efficacy of losartan for treatment of children with RDEB (EudraCT Number: 2015–003670-32, interim results presented in First World Congress on Epidermolysis Bullosa, January 19–23, 2020, London, United Kingdom (2020).

While clear effects on reduction of TGFβ activity were seen in the losartan-treated collagen VII hypomorphic mice, the most prominent was on inflammation (Nyström et al., 2015). Thus, a major benefit of losartan appeared to be from reduction of inflammation.

### Inflammation

Peripheral and systemic alterations in immune cell number and activity have been observed in RDEB (Lazarus, 1972; Tyring et al., 1989; Chopra et al., 1990, 1992). Because RDEB is associated with chronic tissue damage, inflammation could be a consequence of the damage and not involved in modifying disease manifestations. However, different lines of evidence from human and animal models suggest that DEB can be considered a systemic inflammatory disease (Annicchiarico et al., 2015; Nyström et al., 2015; Figure 2).

In collagen VII hypomorphic mice, natural disease severity positively correlated with myeloid cell numbers and immunoglobulin content in fibrotic skin (Nyström et al., 2015). Transcriptomic investigation of biopsies from scarred DEB skin revealed signs of heightened inflammation of DEB skin and posited mitten deformities to be in part inflammation-driven (Breitenbach et al., 2015a, 2015b). There is an element of autoimmunity in DEB. Analyses have revealed presence of antibodies against DEJ proteins including collagen VII in people with RDEB (Tampoia et al., 2013; Woodley et al., 2014; Annicchiarico et al., 2015). The level of autoantibodies and level of interleukin (IL)-6 and−12 showed positive correlation with disease severity in EB and specifically in DEB (Tampoia et al., 2013; Annicchiarico et al., 2015). A subsequent study indicated that the IL-6 to IL-10 ratio in serum could be used as a prognostic marker of disease severity in RDEB (Tampoia et al., 2017).

Direct evidence of inflammation as an active modifier of disease in RDEB is still missing. As suggested by proteomics, the major effect from losartan treatment of RDEB model mice was reduction of inflammation; lower tissue inflammation and lower levels of circulating IL-6 and TGFβ correlated with milder disease. Mesenchymal stromal cells (MSCs) have been of interest for treatment of RDEB, since they, on the one hand, have the ability to produce collagen VII, on the other hand are immunomodulatory (Tolarova and Tolar, 2015). Using the same animal model, we disclosed that the major benefit of MSCs in promoting healing and reducing fibrotic aspects of RDEB wounds was from reduction in tissue inflammation (Kühl et al., 2015). Clinical trials provide a similar picture. It appears that immunosuppression and -modulation after allogeneic bone marrow transplantation, which has been evaluated as a potentially curative treatment for RDEB, confer symptom-relief effects (Wagner et al., 2010; Ebens et al., 2019). Similarly, systemic administration of allogeneic MSCs alone has improved the wellbeing of people with RDEB by modulating inflammation, without increasing collagen VII deposition (Petrof et al., 2015; Maseda et al., 2020). Since RDEB is associated with a heavily heightened risk of developing aggressive SCC, it is important to not apply general immunosuppression but to keep anti-tumor immunity intact. Therefore, more studies are needed to better understand which aspects of inflammation and immunity in RDEB correlate with and potentially drive disease.

### Angiogenesis

While not yet functionally assessed in studies, another potential disease modifier in RDEB could be increased angiogenesis (Arbiser et al., 1998; Martins et al., 2016). As shown in the context of cancer development and growth, angiogenesis is increased in RDEB skin prior to the occurrence of tumors (Martins et al., 2016). Mechanistically, a higher vascularization of the tissue may lead to increased vascular leakage after damage, which could increase the stiffness of the tissue and evoke pro-fibrotic stiffness responses of fibroblasts that, in turn, support tumorigenesis (Figure 2).

### Therapeutic Perspective

Like in many monogenetic diseases, several factors outside the mutational status determine the disease severity and phenotypic presentation of RDEB. With disease progression, the reliance on such factors is likely to change. Because of the rarity of the disease and the large mutational spectra of RDEB, it is challenging to identify factors in detail and on a mechanistic level in the human system. Here, the use of small-animal models greatly facilitates the discovery and functional assessment of disease-modulating mechanisms, as spontaneous and genetically engineered small-animal models with identical with *COL7A1* mutations display diversity in phenotypic presentations (Nyström et al., 2013a, 2015; Smith et al., 2021).

A feared complication of RDEB is the early occurrence of highly aggressive squamous cell carcinoma (Guerra et al., 2017; Cho et al., 2018). Evidence points to that the aggressiveness is in large caused by changes in the microenvironment (Ng et al., 2012; Mittapalli et al., 2016; Guerra et al., 2017). It should be emphasized that all the discussed disease modifiers have been implicated in supporting the progression of SCC in the context of RDEB-associated SCCs and other SCCs (Ng et al., 2012; Martins et al., 2016; Mittapalli et al., 2016; Föll et al., 2017; Rahmati Nezhad et al., 2021). Thus, therapies aiming at modulating the activity of these disease modifiers could create a microenvironment less supportive of malignant conversion of keratinocytes and high-risk tumor behavior.

An improved knowledge of disease-modulating mechanisms in RDEB will facilitate the development of new disease-stage specific, symptom-relief therapies. These could also have potential to improve the efficacy of curative therapies. For example, reducing inflammation and tissue-damaging processes in the dermal microenvironment should improve the take and healing of gene-corrected keratinocyte grafts that have been tested for treatment of EB (Nyström and Bruckner-Tuderman, 2016; Marinkovich and Tang, 2019). Since many of the already known disease-modulating or disease-associated mechanisms in RDEB are naturally active and important for wound healing and tissue regeneration, it is important to carefully test treatment combinations. Furthermore, as RDEB is a life-long and systemic disease, it is essential to consider the nature and actions of potential drug candidates on a system level over an extended time. For example, targeting certain aspects of inflammation, such as strong suppression of adaptive immunity, may reduce fibrosis and itch and improve wound healing but could also increase occurrence of cancers.

The aim of future research in the disease-modulating space of RDEB should be to obtain careful mechanistic knowledge and translate this into evidence-based therapies that will relieve symptoms and slow disease progression.

## Author Contributions

AN, LB-T, and DK: writing original draft and manuscript editing. AN and DK: figure preparation. All authors contributed to the article and approved the submitted version.

## Funding

The listed funders have supported the authors’ research on the discussed topics. The German Research Foundation (DFG) through NY90/5-1, KI1795/2-1, SFB850 project B11, SFB1160 project B03, SFB1479 Project ID: 441891347- P13, a grant from the Fritz Thyssen Stiftung (Az. 10.19.1.016MN) and a grant from EB Research Partnership.

## Conflict of Interest

The authors declare that the research was conducted in the absence of any commercial or financial relationships that could be construed as a potential conflict of interest.

## Publisher’s Note

All claims expressed in this article are solely those of the authors and do not necessarily represent those of their affiliated organizations, or those of the publisher, the editors and the reviewers. Any product that may be evaluated in this article, or claim that may be made by its manufacturer, is not guaranteed or endorsed by the publisher.

## Abbreviations

EB: epidermolysis bullosa
ECM: extracellular matrix
DEB: dystrophic EB
DDEB: dominant DEB
DEJ: dermal-epidermal junction
IL: interleukin
NC: non-collagenous
LH3: lysyl hydroxylase 3
MMP: matrix metalloproteinase
RDEB: recessive DEB
TGF: transforming growth factor

## References

[ref1] Aho S. Uitto J. (1998). Two-hybrid analysis reveals multiple direct interactions for thrombospondin 1. Matrix Biol. 17, 401–412. doi: 10.1016/S0945-053X(98)90100-7, PMID: 9840442

[ref2] Akasaka E. Kleiser S. Sengle G. Bruckner-Tuderman L. Nyström A. (2021). Diversity of mechanisms underlying latent TGF-β activation in recessive dystrophic epidermolysis bullosa. J. Invest. Dermatol. 141, 1450–1460.e9. doi: 10.1016/j.jid.2020.10.024, PMID: 33333127

[ref3] Almaani N. Liu L. Dopping-Hepenstal P. J. C. Lai-Cheong J. E. Wong A. Nanda A.. (2011). Identical glycine substitution mutations in type VII collagen may underlie both dominant and recessive forms of dystrophic epidermolysis bullosa. Acta Derm. Venereol. 91, 262–266. doi: 10.2340/00015555-1053, PMID: 21448560

[ref4] Almaani N. Liu L. Harrison N. Tanaka A. Lai-Cheong J. Mellerio J. E.. (2009). New glycine substitution mutations in type VII collagen underlying epidermolysis bullosa pruriginosa but the phenotype is not explained by a common polymorphism in the matrix metalloproteinase-1 gene promoter. Acta Derm. Venereol. 89, 6–11. doi: 10.2340/00015555-0605, PMID: 19197535

[ref5] Annicchiarico G. Morgese M. G. Esposito S. Lopalco G. Lattarulo M. Tampoia M.. (2015). Proinflammatory cytokines and Antiskin autoantibodies in patients With inherited epidermolysis bullosa. Medicine 94:e1528. doi: 10.1097/MD.0000000000001528, PMID: 26496255 PMC4620762

[ref6] Arbiser J. L. Fine J. D. Murrell D. Paller A. Connors S. Keough K.. (1998). Basic fibroblast growth factor: a missing link between collagen VII, increased collagenase, and squamous cell carcinoma in recessive dystrophic epidermolysis bullosa. Mol. Med. 4, 191–195. doi: 10.1007/BF03401916, PMID: 9562977 PMC2230348

[ref7] Atanasova V. S. Russell R. J. Webster T. G. Cao Q. Agarwal P. Lim Y. Z.. (2019). Thrombospondin-1 is a major activator of TGF-β signaling in recessive dystrophic epidermolysis bullosa fibroblasts. J. Invest. Dermatol. 139, 1497–1505.e5. doi: 10.1016/j.jid.2019.01.011, PMID: 30684555

[ref8] Bar J. Sarig O. Lotan-Pompan M. Dassa B. Miodovnik M. Weinberger A. (2021). Evidence for cutaneous dysbiosis in dystrophic epidermolysis bullosa. Clin. Exp. Dermatol. doi: 10.1111/ced.14592 [Epub ahead of print] 33682945

[ref9] Bardhan A. Bruckner-Tuderman L. Chapple I. L. C. Fine J.-D. Harper N. Has C.. (2020). Epidermolysis bullosa. Nat. Rev. Dis. Primers. 6:78. doi: 10.1038/s41572-020-0210-0, PMID: 32973163

[ref10] Bauer E. A. Cooper T. W. Tucker D. R. Esterly N. B. (1980). Phenytoin therapy of recessive dystrophic epidermolysis bullosa. Clinical trial and proposed mechanism of action on collagenase. N. Engl. J. Med. 303, 776–781. doi: 10.1056/NEJM198010023031402, PMID: 6251365

[ref11] Bauer E. A. Eisen A. Z. (1978). Recessive dystrophic epidermolysis bullosa. Evidence for increased collagenase as a genetic characteristic in cell culture. J. Exp. Med. 148, 1378–1387. doi: 10.1084/jem.148.5.1378, PMID: 214508 PMC2185054

[ref12] Berberich B. Thriene K. Gretzmeier C. Kühl T. Bayer H. Athanasiou I.. (2020). Proteomic profiling of fibroblasts isolated from chronic wounds identifies disease-relevant signaling pathways. J. Invest. Dermatol. 140, 2280–2290.e4. doi: 10.1016/j.jid.2020.02.040, PMID: 32305317

[ref13] Bodemer C. Tchen S. I. Ghomrasseni S. Séguier S. Gaultier F. Fraitag S.. (2003). Skin expression of metalloproteinases and tissue inhibitor of metalloproteinases in sibling patients with recessive dystrophic epidermolysis and intrafamilial phenotypic variation. J. Invest. Dermatol. 121, 273–279. doi: 10.1046/j.1523-1747.2003.12325.x, PMID: 12880418

[ref14] Breitenbach J. Gruber C. Klausegger A. Trost A. Bogner B. Reitsamer H.. (2015a). Pseudosyndactyly - an inflammatory and fibrotic wound healing disorder in recessive dystrophic epidermolysis bullosa. J. Dtsch. Dermatol. Ges. 13, 1257–1266. doi: 10.1111/ddg.12839, PMID: 26612796

[ref15] Breitenbach J. S. Rinnerthaler M. Trost A. Weber M. Klausegger A. Gruber C.. (2015b). Transcriptome and ultrastructural changes in dystrophic epidermolysis bullosa resemble skin aging. Aging 7, 389–411. doi: 10.18632/aging.100755, PMID: 26143532 PMC4505166

[ref16] Brittingham R. Uitto J. Fertala A. (2006). High-affinity binding of the NC1 domain of collagen VII to laminin 5 and collagen IV. Biochem. Biophys. Res. Commun. 343, 692–699. doi: 10.1016/j.bbrc.2006.03.034, PMID: 16563355

[ref17] Brown J. R. Milgraum D. M. Riyaz F. R. Jahnke M. N. Thottam P. J. (2017). Successful placement of a BAHA implant in a patient With epidermolysis bullosa: a case report and review of the literature. Ann. Otol. Rhinol. Laryngol. 126, 778–780. doi: 10.1177/0003489417729833, PMID: 28895441

[ref18] Bruckner-Tuderman L. Nilssen O. Zimmermann D. R. Dours-Zimmermann M. T. Kalinke D. U. Gedde-Dahl T.. (1995). Immunohistochemical and mutation analyses demonstrate that procollagen VII is processed to collagen VII through removal of the NC-2 domain. J. Cell Biol. 131, 551–559. doi: 10.1083/jcb.131.2.551, PMID: 7593178 PMC2199977

[ref19] Caldwell-Brown D. Stern R. S. Lin A. N. Carter D. M. (1992). Lack of efficacy of phenytoin in recessive dystrophic epidermolysis bullosa. Epidermolysis bullosa study group. N. Engl. J. Med. 327, 163–167. doi: 10.1056/NEJM199207163270305 1608407

[ref20] Chacón-Solano E. León C. Díaz F. García-García F. García M. Escámez M. J.. (2019). Fibroblast activation and abnormal extracellular matrix remodelling as common hallmarks in three cancer-prone genodermatoses. Br. J. Dermatol. 181, 512–522. doi: 10.1111/bjd.17698, PMID: 30693469 PMC6850467

[ref21] Chen M. Keene D. R. Costa F. K. Tahk S. H. Woodley D. T. (2001). The carboxyl terminus of type VII collagen mediates antiparallel dimer formation and constitutes a new antigenic epitope for epidermolysis bullosa acquisita autoantibodies. J. Biol. Chem. 276, 21649–21655. doi: 10.1074/jbc.M100180200, PMID: 11274208

[ref22] Chen M. Marinkovich M. P. Jones J. C. O’Toole E. A. Li Y. Y. Woodley D. T. (1999). NC1 domain of type VII collagen binds to the beta3 chain of laminin 5 via a unique subdomain within the fibronectin-like repeats. J. Invest. Dermatol. 112, 177–183. doi: 10.1046/j.1523-1747.1999.00491.x, PMID: 9989793

[ref23] Chen M. Marinkovich M. P. Veis A. Cai X. Rao C. N. O’Toole E. A.. (1997). Interactions of the amino-terminal noncollagenous (NC1) domain of type VII collagen with extracellular matrix components. A potential role in epidermal-dermal adherence in human skin. J. Biol. Chem. 272, 14516–14522. doi: 10.1074/jbc.272.23.14516, PMID: 9169408

[ref24] Cho R. J. Alexandrov L. B. den Breems N. Y. Atanasova V. S. Farshchian M. Purdom E.. (2018). APOBEC mutation drives early-onset squamous cell carcinomas in recessive dystrophic epidermolysis bullosa. Sci. Transl. Med. 10:eaas9668. doi: 10.1126/scitranslmed.aas9668, PMID: 30135250

[ref25] Chopra V. Tyring S. K. Johnson L. Fine J. D. (1990). Patients with severe forms of inherited epidermolysis bullosa exhibit decreased lymphokine and monokine production. J. Clin. Immunol. 10, 321–329. doi: 10.1007/BF00917477, PMID: 2128088

[ref26] Chopra V. Tyring S. K. Johnson L. Fine J. D. (1992). Peripheral blood mononuclear cell subsets in patients with severe inherited forms of epidermolysis bullosa. Arch. Dermatol. 128, 201–209. doi: 10.1001/archderm.1992.01680120073006, PMID: 1739298

[ref27] Chung H. J. Uitto J. (2010). Type VII collagen: the anchoring fibril protein at fault in dystrophic epidermolysis bullosa. Dermatol. Clin. 28, 93–105. doi: 10.1016/j.det.2009.10.011, PMID: 19945621 PMC2791403

[ref28] Cianfarani F. De Domenico E. Nyström A. Mastroeni S. Abeni D. Baldini E.. (2019). Decorin counteracts disease progression in mice with recessive dystrophic epidermolysis bullosa. Matrix Biol. 81, 3–16. doi: 10.1016/j.matbio.2018.12.001, PMID: 30528862

[ref29] COL7A1(gene) - ClinVar – NCBI (2020). Available at: https://www.ncbi.nlm.nih.gov/clinvar/?term=COL7A1%5Bgene%5D (Accessed August 25, 2021).

[ref30] Condorelli A. G. Dellambra E. Logli E. Zambruno G. Castiglia D. (2019). Epidermolysis bullosa-associated squamous cell carcinoma: From pathogenesis to therapeutic perspectives. Int. J. Mol. Sci. 20:5707. doi: 10.3390/ijms20225707, PMID: 31739489 PMC6888002

[ref31] Dang N. Murrell D. F. (2008). Mutation analysis and characterization of COL7A1 mutations in dystrophic epidermolysis bullosa. Exp. Dermatol. 17, 553–568. doi: 10.1111/j.1600-0625.2008.00723.x, PMID: 18558993

[ref33] Ebens C. L. McGrath J. A. Tamai K. Hovnanian A. Wagner J. E. Riddle M. J.. (2019). Bone marrow transplant with post-transplant cyclophosphamide for recessive dystrophic epidermolysis bullosa expands the related donor pool and permits tolerance of nonhaematopoietic cellular grafts. Br. J. Dermatol. 181, 1238–1246. doi: 10.1111/bjd.17858, PMID: 30843184 PMC6731170

[ref34] Eisen A. Z. (1969). Human skin collagenase: relationship to the pathogenesis of epidermolysis bullosa dystrophica. J. Invest. Dermatol. 52, 449–453. doi: 10.1038/jid.1969.77, PMID: 4305996

[ref35] Esposito S. Guez S. Orenti A. Tadini G. Scuvera G. Corti L.. (2016). Autoimmunity and cytokine imbalance in inherited epidermolysis bullosa. Int. J. Mol. Sci. 17:1625. doi: 10.3390/ijms17101625, PMID: 27669234 PMC5085658

[ref36] Fields G. B. (2019). The rebirth of matrix metalloproteinase inhibitors: moving Beyond the dogma. Cell 8:984. doi: 10.3390/cells8090984, PMID: 31461880 PMC6769477

[ref37] Figueira E. C. Murrell D. F. Coroneo M. T. (2010). Ophthalmic involvement in inherited epidermolysis bullosa. Dermatol. Clin. 28, 143–152. doi: 10.1016/j.det.2009.10.021, PMID: 19945628

[ref38] Fine J.-D. Hall M. Weiner M. Li K.-P. Suchindran C. (2008). The risk of cardiomyopathy in inherited epidermolysis bullosa. Br. J. Dermatol. 159, 677–682. doi: 10.1111/j.1365-2133.2008.08697.x, PMID: 18616785 PMC2592258

[ref39] Fine J.-D. Johnson L. B. Weiner M. Li K.-P. Suchindran C. (2009). Epidermolysis bullosa and the risk of life-threatening cancers: the national EB registry experience, 1986-2006. J. Am. Acad. Dermatol. 60, 203–211. doi: 10.1016/j.jaad.2008.09.035, PMID: 19026465

[ref40] Föll M. C. Fahrner M. Gretzmeier C. Thoma K. Biniossek M. L. Kiritsi D.. (2017). Identification of tissue damage, extracellular matrix remodeling and bacterial challenge as common mechanisms associated with high-risk cutaneous squamous cell carcinomas. Matrix Biol. 66, 1–21. doi: 10.1016/j.matbio.2017.11.004, PMID: 29158163

[ref41] Fritsch A. Loeckermann S. Kern J. S. Braun A. Bösl M. R. Bley T. A.. (2008). A hypomorphic mouse model of dystrophic epidermolysis bullosa reveals mechanisms of disease and response to fibroblast therapy. J. Clin. Invest. 118, 1669–1679. doi: 10.1172/JCI34292, PMID: 18382769 PMC2276400

[ref42] Fuentes I. Guttmann-Gruber C. Tay A. S. L. Piñón Hofbauer J. Denil S. L. I. J. Reichelt J.. (2018). Reduced microbial diversity is a feature of recessive dystrophic epidermolysis bullosa-involved skin and wounds. J. Invest. Dermatol. 138, 2492–2495. doi: 10.1016/j.jid.2018.04.026, PMID: 29753707

[ref43] Guerra L. Odorisio T. Zambruno G. Castiglia D. (2017). Stromal microenvironment in type VII collagen-deficient skin: the ground for squamous cell carcinoma development. Matrix Biol. 63, 1–10. doi: 10.1016/j.matbio.2017.01.002, PMID: 28126522

[ref44] Habashi J. P. Judge D. P. Holm T. M. Cohn R. D. Loeys B. L. Cooper T. K.. (2006). Losartan, an AT1 antagonist, prevents aortic aneurysm in a mouse model of Marfan syndrome. Science 312, 117–121. doi: 10.1126/science.1124287, PMID: 16601194 PMC1482474

[ref45] Has C. Bauer J. W. Bodemer C. Bolling M. C. Bruckner-Tuderman L. Diem A.. (2020). Consensus reclassification of inherited epidermolysis bullosa and other disorders with skin fragility. Br. J. Dermatol. 183, 614–627. doi: 10.1111/bjd.18921, PMID: 32017015

[ref46] Has C. Nyström A. Saeidian A. H. Bruckner-Tuderman L. Uitto J. (2018). Epidermolysis bullosa: molecular pathology of connective tissue components in the cutaneous basement membrane zone. Matrix Biol. 72, 313–329. doi: 10.1016/j.matbio.2018.04.001, PMID: 29627521

[ref47] Hinz B. (2015). The extracellular matrix and transforming growth factor-β1: tale of a strained relationship. Matrix Biol. 47, 54–65. doi: 10.1016/j.matbio.2015.05.006, PMID: 25960420

[ref48] Hoste E. Arwert E. N. Lal R. South A. P. Salas-Alanis J. C. Murrell D. F.. (2015). Innate sensing of microbial products promotes wound-induced skin cancer. Nat. Commun. 6:5932. doi: 10.1038/ncomms6932, PMID: 25575023 PMC4338544

[ref49] Hovnanian A. Rochat A. Bodemer C. Petit E. Rivers C. A. Prost C.. (1997). Characterization of 18 new mutations in COL7A1 in recessive dystrophic epidermolysis bullosa provides evidence for distinct molecular mechanisms underlying defective anchoring fibril formation. Am. J. Hum. Genet. 61, 599–610. doi: 10.1086/515495, PMID: 9326325 PMC1715975

[ref50] Järvinen T. A. H. Ruoslahti E. (2010). Target-seeking antifibrotic compound enhances wound healing and suppresses scar formation in mice. Proc. Natl. Acad. Sci. U. S. A. 107, 21671–21676. doi: 10.1073/pnas.1016233107, PMID: 21106754 PMC3003105

[ref51] Kern J. S. Grüninger G. Imsak R. Müller M. L. Schumann H. Kiritsi D.. (2009). Forty-two novel COL7A1 mutations and the role of a frequent single nucleotide polymorphism in the MMP1 promoter in modulation of disease severity in a large European dystrophic epidermolysis bullosa cohort. Br. J. Dermatol. 161, 1089–1097. doi: 10.1111/j.1365-2133.2009.09333.x, PMID: 19681861

[ref52] Kim K. K. Sheppard D. Chapman H. A. (2018). TGF-β1 signaling and tissue fibrosis. Cold Spring Harb. Perspect. Biol. 10:a022293. doi: 10.1101/cshperspect.a022293, PMID: 28432134 PMC5880172

[ref53] Kim W. B. Alavi A. Walsh S. Kim S. Pope E. (2015). Epidermolysis bullosa pruriginosa: a systematic review exploring genotype-phenotype correlation. Am. J. Clin. Dermatol. 16, 81–87. doi: 10.1007/s40257-015-0119-7, PMID: 25690953

[ref32] Kiritsi D. (2020). EB20200 first world congress on epidermolysis bullosa, January 19–23, London, United Kingdom. Acta Dermato-Venereologica. 100, 7–7. doi: 10.2340/00015555-3586 PMC11262388 32617601

[ref54] Kiritsi D. Nyström A. (2018). The role of TGFβ in wound healing pathologies. Mech. Ageing Dev. 172, 51–58. doi: 10.1016/j.mad.2017.11.004, PMID: 29132871

[ref55] Knaup J. Gruber C. Krammer B. Ziegler V. Bauer J. Verwanger T. (2011). TGFβ-signaling in squamous cell carcinoma occurring in recessive dystrophic epidermolysis bullosa. Anal. Cell. Pathol. 34, 339–353. doi: 10.1155/2011/153108, PMID: 22002724 PMC4605790

[ref56] Krämer S. Lucas J. Gamboa F. Peñarrocha Diago M. Peñarrocha Oltra D. Guzmán-Letelier M.. (2020). Clinical practice guidelines: Oral health care for children and adults living with epidermolysis bullosa. Spec. Care Dentist. 40(Suppl. 1), 3–81. doi: 10.1111/scd.12511, PMID: 33202040 PMC7756753

[ref57] Kruppa D. Peters F. Bornert O. Maler M. D. Martin S. F. Becker-Pauly C.. (2021). Distinct contributions of meprins to skin regeneration after injury – meprin α a physiological processer of pro-collagen VII. Matrix Biology Plus 11:100065. doi: 10.1016/j.mbplus.2021.100065, PMID: 34435182 PMC8377016

[ref58] Kühl T. Mezger M. Hausser I. Handgretinger R. Bruckner-Tuderman L. Nyström A. (2015). High local concentrations of intradermal MSCs restore skin integrity and facilitate wound healing in dystrophic epidermolysis bullosa. Mol. Ther. 23, 1368–1379. doi: 10.1038/mt.2015.58, PMID: 25858020 PMC4817872

[ref59] Küttner V. Mack C. Gretzmeier C. Bruckner-Tuderman L. Dengjel J. (2014). Loss of collagen VII is associated with reduced transglutaminase 2 abundance and activity. J. Invest. Dermatol. 134, 2381–2389. doi: 10.1038/jid.2014.185, PMID: 24732400

[ref60] Küttner V. Mack C. Rigbolt K. T. G. Kern J. S. Schilling O. Busch H.. (2013). Global remodelling of cellular microenvironment due to loss of collagen VII. Mol. Syst. Biol. 9:657. doi: 10.1038/msb.2013.17, PMID: 23591773 PMC3658272

[ref61] Lazarus G. S. (1972). Collagenase and connective tissue metabolism in epidermolysis bullosa. J. Invest. Dermatol. 58, 242–248. doi: 10.1111/1523-1747.ep12539946, PMID: 4336522

[ref62] Levin L. E. Shayegan L. H. Lucky A. W. Hook K. P. Bruckner A. L. Feinstein J. A.. (2021). Characterization of wound microbes in epidermolysis bullosa: results from the epidermolysis bullosa clinical characterization and outcomes database. Pediatr. Dermatol. 38, 119–124. doi: 10.1111/pde.14444, PMID: 33247481 PMC7906915

[ref63] Liao Y. Ivanova L. Zhu H. Plumer T. Hamby C. Mehta B.. (2018). Cord blood-derived stem cells suppress fibrosis and May prevent malignant progression in recessive dystrophic epidermolysis bullosa. Stem Cells 36, 1839–1850. doi: 10.1002/stem.2907, PMID: 30247783

[ref64] Marinkovich M. P. Tang J. Y. (2019). Gene therapy for epidermolysis bullosa. J. Invest. Dermatol. 139, 1221–1226. doi: 10.1016/j.jid.2018.11.036, PMID: 31068252

[ref65] Martins V. L. Caley M. P. Moore K. Szentpetery Z. Marsh S. T. Murrell D. F.. (2016). Suppression of TGFβ and angiogenesis by type VII collagen in cutaneous SCC. J. Natl. Cancer Inst. 108:djv293. doi: 10.1093/jnci/djv293, PMID: 26476432

[ref66] Maseda R. Martínez-Santamaría L. Sacedón R. Butta N. de Arriba M. D. C. García-Barcenilla S.. (2020). Beneficial effect of systemic allogeneic adipose derived mesenchymal cells on the clinical, inflammatory and immunologic status of a patient With recessive dystrophic epidermolysis bullosa: a case report. Front. Med. 7:576558. doi: 10.3389/fmed.2020.576558, PMID: 33324660 PMC7726418

[ref67] Mittapalli V. R. Madl J. Löffek S. Kiritsi D. Kern J. S. Römer W.. (2016). Injury-driven stiffening of the dermis expedites skin carcinoma progression. Cancer Res. 76, 940–951. doi: 10.1158/0008-5472.CAN-15-1348, PMID: 26676755

[ref68] Moali C. Font B. Ruggiero F. Eichenberger D. Rousselle P. François V.. (2005). Substrate-specific modulation of a multisubstrate proteinase. C-terminal processing of fibrillar procollagens is the only BMP-1-dependent activity to be enhanced by PCPE-1. J. Biol. Chem. 280, 24188–24194. doi: 10.1074/jbc.M501486200, PMID: 15834133

[ref69] Montaudié H. Chiaverini C. Sbidian E. Charlesworth A. Lacour J.-P. (2016). Inherited epidermolysis bullosa and squamous cell carcinoma: a systematic review of 117 cases. Orphanet J. Rare Dis. 11:117. doi: 10.1186/s13023-016-0489-9, PMID: 27544590 PMC4992553

[ref70] Neill T. Schaefer L. Iozzo R. V. (2012). Decorin: a guardian from the matrix. Am. J. Pathol. 181:22735579, 380–387. doi: 10.1016/j.ajpath.2012.04.029 PMC3409438 22735579

[ref71] Ng Y.-Z. Pourreyron C. Salas-Alanis J. C. Dayal J. H. S. Cepeda-Valdes R. Yan W.. (2012). Fibroblast-derived dermal matrix drives development of aggressive cutaneous squamous cell carcinoma in patients with recessive dystrophic epidermolysis bullosa. Cancer Res. 72, 3522–3534. doi: 10.1158/0008-5472.CAN-11-2996, PMID: 22564523

[ref72] Nyström A. Bornert O. Kühl T. Gretzmeier C. Thriene K. Dengjel J.. (2018). Impaired lymphoid extracellular matrix impedes antibacterial immunity in epidermolysis bullosa. Proc. Natl. Acad. Sci. U. S. A. 115, E705–E714. doi: 10.1073/pnas.1709111115, PMID: 29305555 PMC5789908

[ref73] Nyström A. Bruckner-Tuderman L. (2016). Gene therapy for epidermolysis bullosa: sticky business. Mol. Ther. 24, 2035–2036. doi: 10.1038/mt.2016.199, PMID: 27966558 PMC5167791

[ref74] Nyström A. Buttgereit J. Bader M. Shmidt T. Ozcelik C. Hausser I.. (2013a). Rat model for dominant dystrophic epidermolysis bullosa: glycine substitution reduces collagen VII stability and shows gene-dosage effect. PLoS One 8:e64243. doi: 10.1371/journal.pone.0064243, PMID: 23717576 PMC3662756

[ref75] Nyström A. Thriene K. Mittapalli V. Kern J. S. Kiritsi D. Dengjel J.. (2015). Losartan ameliorates dystrophic epidermolysis bullosa and uncovers new disease mechanisms. EMBO Mol. Med. 7, 1211–1228. doi: 10.15252/emmm.201505061, PMID: 26194911 PMC4568953

[ref76] Nyström A. Velati D. Mittapalli V. R. Fritsch A. Kern J. S. Bruckner-Tuderman L. (2013b). Collagen VII plays a dual role in wound healing. J. Clin. Invest. 123, 3498–3509. doi: 10.1172/JCI68127, PMID: 23867500 PMC3726167

[ref77] Odorisio T. Di Salvio M. Orecchia A. Di Zenzo G. Piccinni E. Cianfarani F.. (2014). Monozygotic twins discordant for recessive dystrophic epidermolysis bullosa phenotype highlight the role of TGF-β signalling in modifying disease severity. Hum. Mol. Genet. 23, 3907–3922. doi: 10.1093/hmg/ddu102, PMID: 24599399

[ref78] Pemmari T. Ivanova L. May U. Lingasamy P. Tobi A. Pasternack A.. (2020). Exposed CendR domain in homing peptide yields skin-targeted therapeutic in epidermolysis bullosa. Mol. Ther. 28, 1833–1845. doi: 10.1016/j.ymthe.2020.05.017, PMID: 32497513 PMC7403337

[ref79] Petrof G. Lwin S. M. Martinez-Queipo M. Abdul-Wahab A. Tso S. Mellerio J. E.. (2015). Potential of systemic allogeneic mesenchymal stromal cell therapy for children with recessive dystrophic epidermolysis bullosa. J. Invest. Dermatol. 135, 2319–2321. doi: 10.1038/jid.2015.158, PMID: 25905587 PMC5696540

[ref80] Py B. F. Gonzalez S. F. Long K. Kim M.-S. Kim Y.-A. Zhu H.. (2013). Cochlin produced by follicular dendritic cells promotes antibacterial innate immunity. Immunity 38, 1063–1072. doi: 10.1016/j.immuni.2013.01.015, PMID: 23684986 PMC3758559

[ref81] Raghunath M. Höpfner B. Aeschlimann D. Lüthi U. Meuli M. Altermatt S.. (1996). Cross-linking of the dermo-epidermal junction of skin regenerating from keratinocyte autografts. Anchoring fibrils are a target for tissue transglutaminase. J. Clin. Invest. 98, 1174–1184. doi: 10.1172/JCI118901, PMID: 8787681 PMC507540

[ref82] Rahmati Nezhad P. Riihilä P. Piipponen M. Kallajoki M. Meri S. Nissinen L.. (2021). Complement factor I upregulates expression of matrix metalloproteinase-13 and -2 and promotes invasion of cutaneous squamous carcinoma cells. Exp. Dermatol. doi: 10.1111/exd.14349 [Epub ahead of print] 33813765

[ref83] Ramirez H. Patel S. B. Pastar I. (2014). The role of TGFβ signaling in wound epithelialization. Adv. Wound Care 3, 482–491. doi: 10.1089/wound.2013.0466, PMID: 25032068 PMC4086377

[ref84] Rattenholl A. Pappano W. N. Koch M. Keene D. R. Kadler K. E. Sasaki T.. (2002). Proteinases of the bone morphogenetic protein-1 family convert procollagen VII to mature anchoring fibril collagen. J. Biol. Chem. 277, 26372–26378. doi: 10.1074/jbc.M203247200, PMID: 11986329

[ref85] Reimer A. Hess M. Schwieger-Briel A. Kiritsi D. Schauer F. Schumann H.. (2020). Natural history of growth and anaemia in children with epidermolysis bullosa: a retrospective cohort study. Br. J. Dermatol. 182, 1437–1448. doi: 10.1111/bjd.18475, PMID: 31487386

[ref86] Risteli M. Ruotsalainen H. Salo A. M. Sormunen R. Sipilä L. Baker N. L.. (2009). Reduction of lysyl hydroxylase 3 causes deleterious changes in the deposition and organization of extracellular matrix. J. Biol. Chem. 284, 28204–28211. doi: 10.1074/jbc.M109.038190, PMID: 19696018 PMC2788872

[ref87] Ryynänen J. Sollberg S. Olsen D. R. Uitto J. (1991). Transforming growth factor-beta up-regulates type VII collagen gene expression in normal and transformed epidermal keratinocytes in culture. Biochem. Biophys. Res. Commun. 180, 673–680. doi: 10.1016/S0006-291X(05)81118-0, PMID: 1953739

[ref88] Salmon H. Franciszkiewicz K. Damotte D. Dieu-Nosjean M.-C. Validire P. Trautmann A.. (2012). Matrix architecture defines the preferential localization and migration of T cells into the stroma of human lung tumors. J. Clin. Invest. 122, 899–910. doi: 10.1172/JCI45817, PMID: 22293174 PMC3287213

[ref89] Salo A. M. Cox H. Farndon P. Moss C. Grindulis H. Risteli M.. (2008). A connective tissue disorder caused by mutations of the lysyl hydroxylase 3 gene. Am. J. Hum. Genet. 83, 495–503. doi: 10.1016/j.ajhg.2008.09.004, PMID: 18834968 PMC2561927

[ref90] Salo A. M. Wang C. Sipilä L. Sormunen R. Vapola M. Kervinen P.. (2006). Lysyl hydroxylase 3 (LH3) modifies proteins in the extracellular space, a novel mechanism for matrix remodeling. J. Cell. Physiol. 207, 644–653. doi: 10.1002/jcp.20596, PMID: 16447251

[ref91] Sanjabi S. Oh S. A. Li M. O. (2017). Regulation of the immune response by TGF-β: From conception to autoimmunity and infection. Cold Spring Harb. Perspect. Biol. 9:a022236. doi: 10.1101/cshperspect.a022236, PMID: 28108486 PMC5453394

[ref92] Smith B. R. C. Nystrom A. Nowell C. J. Hausser I. Gretzmeier C. Robertson S. J.. (2021). Mouse models for dominant dystrophic epidermolysis bullosa (DDEB) carrying common human point mutations recapitulate the human disease. Dis. Model. Mech. 14:dmm048082. doi: 10.1242/dmm.048082, PMID: 34085701 PMC8214732

[ref93] Solis D. C. Gorell E. S. Teng C. Barriga M. Nazaroff J. Li S.. (2021). Clinical characteristics associated with increased wound size in patients with recessive dystrophic epidermolysis bullosa. Pediatr. Dermatol. 38, 704–706. doi: 10.1111/pde.14576, PMID: 33749033

[ref94] Supp D. M. Hahn J. M. Combs K. A. McFarland K. L. Schwentker A. Boissy R. E.. (2019). Collagen VII expression is required in Both keratinocytes and fibroblasts for anchoring fibril formation in bilayer engineered skin substitutes. Cell Transplant. 28, 1242–1256. doi: 10.1177/0963689719857657, PMID: 31271052 PMC6767893

[ref95] Tampoia M. Abbracciavento L. Morrone M. Fumarulo R. (2017). IL-6/IL-10 ratio as A prognostic and predictive marker of the severity of inherited epidermolysis bullosa. Iran. J. Immunol. 14, 340–349. PMID: . 29276186 10.22034/iji.2017.39329

[ref96] Tampoia M. Bonamonte D. Filoni A. Garofalo L. Morgese M. G. Brunetti L.. (2013). Prevalence of specific anti-skin autoantibodies in a cohort of patients with inherited epidermolysis bullosa. Orphanet J. Rare Dis. 8:132. doi: 10.1186/1750-1172-8-132, PMID: 24007552 PMC4015699

[ref97] Thriene K. Grüning B. A. Bornert O. Erxleben A. Leppert J. Athanasiou I.. (2018). Combinatorial omics analysis reveals perturbed lysosomal homeostasis in collagen VII-deficient keratinocytes. *Mol*. Cell Proteomics 17, 565–579. doi: 10.1074/mcp.RA117.000437, PMID: 29326176 PMC5880109

[ref98] Titeux M. Pendaries V. Tonasso L. Décha A. Bodemer C. Hovnanian A. (2008). A frequent functional SNP in the MMP1 promoter is associated with higher disease severity in recessive dystrophic epidermolysis bullosa. Hum. Mutat. 29, 267–276. doi: 10.1002/humu.20647, PMID: 18030675

[ref99] Tolarova M. Tolar J. (2015). From mesoderm to Mesodermatology: bone marrow mesenchymal cells heal skin wounds. Mol. Ther. 23, 1283–1284. doi: 10.1038/mt.2015.84, PMID: 26227251 PMC4817871

[ref100] Twaroski K. Eide C. Riddle M. J. Xia L. Lees C. J. Chen W.. (2019). Revertant mosaic fibroblasts in recessive dystrophic epidermolysis bullosa. Br. J. Dermatol. 181, 1247–1253. doi: 10.1111/bjd.17943, PMID: 30924923 PMC6766431

[ref101] Tyring S. K. Chopra V. Johnson L. Fine J. D. (1989). Natural killer cell activity is reduced in patients with severe forms of inherited epidermolysis bullosa. Arch. Dermatol. 125, 797–800. doi: 10.1001/archderm.1989.01670180069008, PMID: 2730100

[ref102] Vahidnezhad H. Youssefian L. Saeidian A. H. Touati A. Pajouhanfar S. Baghdadi T.. (2019). Mutations in PLOD3, encoding lysyl hydroxylase 3, cause a complex connective tissue disorder including recessive dystrophic epidermolysis bullosa-like blistering phenotype with abnormal anchoring fibrils and type VII collagen deficiency. Matrix Biol. 81, 91–106. doi: 10.1016/j.matbio.2018.11.006, PMID: 30463024

[ref103] Valle K. J. Bauer E. A. (1980). Enhanced biosynthesis of human skin collagenase in fibroblast cultures from recessive dystrophic epidermolysis bullosa. J. Clin. Invest. 66, 176–187. doi: 10.1172/JCI109842, PMID: 6249847 PMC371696

[ref104] van den Akker P. C. Mellerio J. E. Martinez A. E. Liu L. Meijer R. Dopping-Hepenstal P. J. C.. (2011). The inversa type of recessive dystrophic epidermolysis bullosa is caused by specific arginine and glycine substitutions in type VII collagen. J. Med. Genet. 48, 160–167. doi: 10.1136/jmg.2010.082230, PMID: 21113014

[ref105] van den Akker P. C. van Essen A. J. Kraak M. M. J. Meijer R. Nijenhuis M. Meijer G.. (2009). Long-term follow-up of patients with recessive dystrophic epidermolysis bullosa in the Netherlands: expansion of the mutation database and unusual phenotype-genotype correlations. J. Dermatol. Sci. 56, 9–18. doi: 10.1016/j.jdermsci.2009.06.015, PMID: 19665875

[ref106] Villone D. Fritsch A. Koch M. Bruckner-Tuderman L. Hansen U. Bruckner P. (2008). Supramolecular interactions in the dermo-epidermal junction zone: anchoring fibril-collagen VII tightly binds to banded collagen fibrils. J. Biol. Chem. 283, 24506–24513. doi: 10.1074/jbc.M802415200, PMID: 18599485 PMC3259843

[ref107] Wagner J. E. Ishida-Yamamoto A. McGrath J. A. Hordinsky M. Keene D. R. Woodley D. T.. (2010). Bone marrow transplantation for recessive dystrophic epidermolysis bullosa. N. Engl. J. Med. 363, 629–639. doi: 10.1056/NEJMoa0910501, PMID: 20818854 PMC2967187

[ref108] Wang J. Dong X. Zhao B. Li J. Lu C. Springer T. A. (2017). Atypical interactions of integrin αVβ8 with pro-TGF-β1. Proc. Natl. Acad. Sci. U. S. A. 114, E4168–E4174. doi: 10.1073/pnas.1705129114, PMID: 28484027 PMC5448207

[ref109] Wang R. Zhu J. Dong X. Shi M. Lu C. Springer T. A. (2012). GARP regulates the bioavailability and activation of TGFβ. Mol. Biol. Cell 23, 1129–1139. doi: 10.1091/mbc.E11-12-1018, PMID: 22278742 PMC3302739

[ref110] Wang X. Ghasri P. Amir M. Hwang B. Hou Y. Khilili M.. (2013). Topical application of recombinant type VII collagen incorporates Into the dermal-epidermal junction and promotes wound closure. Mol. Ther. 21, 1335–1344. doi: 10.1038/mt.2013.87, PMID: 23670575 PMC3704128

[ref111] Watt S. A. Dayal J. H. S. Wright S. Riddle M. Pourreyron C. McMillan J. R.. (2015). Lysyl hydroxylase 3 localizes to epidermal basement membrane and is reduced in patients with recessive dystrophic epidermolysis bullosa. PLoS One 10:e0137639. doi: 10.1371/journal.pone.0137639, PMID: 26380979 PMC4575209

[ref112] Winberg J. O. Gedde-Dahl T. Bauer E. A. (1989). Collagenase expression in skin fibroblasts from families with recessive dystrophic epidermolysis bullosa. J. Invest. Dermatol. 92, 82–85. doi: 10.1111/1523-1747.ep13071274, PMID: 2535863

[ref113] Winer A. Adams S. Mignatti P. (2018). Matrix metalloproteinase inhibitors in cancer therapy: turning past failures Into future successes. Mol. Cancer Ther. 17, 1147–1155. doi: 10.1158/1535-7163.MCT-17-0646, PMID: 29735645 PMC5984693

[ref114] Woodley D. T. Cogan J. Wang X. Hou Y. Haghighian C. Kudo G.. (2014). De novo anti-type VII collagen antibodies in patients with recessive dystrophic epidermolysis bullosa. J. Invest. Dermatol. 134, 1138–1140. doi: 10.1038/jid.2013.475, PMID: 24213372 PMC3961494

[ref115] Yamaguchi Y. Mann D. M. Ruoslahti E. (1990). Negative regulation of transforming growth factor-beta by the proteoglycan decorin. Nature 346, 281–284. doi: 10.1038/346281a0, PMID: 2374594

